# IMPACTS OF CHANGE FROM 27 TO 30 CHRONIC CONDITIONS ALGORITHM ON PREVALENCE OF DIABETES AND DEMENTIA

**DOI:** 10.1093/geroni/igad104.2913

**Published:** 2023-12-21

**Authors:** Hyosin Kim, Anum Zafar, Haiqun Lin, Soko Setoguchi, Olga Jarrín

**Affiliations:** Oregon State University, Corvallis, Oregon, United States; Rutgers, The State University of New Jersey, New Brunswick, New Jersey, United States; Rutgers, The State University of New Jersey, Newark, New Jersey, United States; Rutgers, The State University of New Jersey, New Brunswick, New Jersey, United States; Rutgers, The State University of New Jersey, New Brunswick, New Jersey, United States

## Abstract

The Centers for Medicare and Medicaid Services (CMS) Chronic Conditions Warehouse (CCW) algorithm used to identify the presence of chronic conditions recently changed. Not only was the number of chronic conditions changed, but also the diagnosis codes and look-back period. Accompanying documentation encourages researchers to empirically assess the change in algorithm for their studies. The purpose of this study is to highlight the impacts of switching to the 30 CCW Chronic Conditions file on the prevalence of selected chronic conditions (diabetes and dementia) in an end-of-life cohort of Medicare beneficiaries (n=2,182,092), with sub-analysis by insurance type. Data sources included the Master Beneficiary Summary File base file and chronic conditions segments (27 vs. 30). The study population was all U.S. Medicare beneficiaries over age 18 who died in 2019. This retrospective observational study summarizes the change in prevalence of diabetes, dementia, and stroke/transient ischemic attack resulting from the new chronic conditions algorithm (27 to 30), stratified by insurance type. Among Medicare beneficiaries who died in 2019, using the 30 vs. 27 Chronic Conditions algorithm decreased the prevalence of diabetes (45% vs. 32%) and dementia (41% vs. 31%). This change may pose challenges for longitudinal and end-of-life research. A decrease in the counting of dementia, diabetes, and stroke cases could lead to an underestimation of their burden on society and be misleading for policymakers and the public. This could result in inadequate policies and resources to address prevention and treatment, which could result in inadequate healthcare services for the affected population.

